# Disparities in *Perimyotis subflavus* Body Mass Between Cave and Culvert Hibernacula in Georgia, USA

**DOI:** 10.1002/ece3.70634

**Published:** 2024-12-06

**Authors:** Emily A. Ferrall, Santiago Perea, Katrina M. Morris, Pete E. Pattavina, Brian J. Irwin, Jeff Hepinstall‐Cymerman, Steven B. Castleberry

**Affiliations:** ^1^ Warnell School of Forestry and Natural Resources University of Georgia Athens Georgia USA; ^2^ Wildlife Conservation Section Georgia Department of Natural Resources Social Circle Georgia USA; ^3^ United States Fish and Wildlife Service, Ecological Services Athens Georgia USA; ^4^ U.S. Geological Survey, Georgia Cooperative Fish & Wildlife Unit, Warnell School of Forestry and Natural Resources University of Georgia Athens Georgia USA

**Keywords:** body mass, cave, culvert, hibernation, *Perimyotis subflavus*, tricolored bat, white‐nose syndrome

## Abstract

The tricolored bat ( *Perimyotis subflavus* ), once common in the eastern United States, has experienced significant mortality due to white‐nose syndrome (WNS), a fungal disease that primarily affects bats hibernating in caves and mines. In coastal regions of the southeastern United States, where caves and mines are scarce, tricolored bats often use roadway culverts as hibernacula. However, WNS infection dynamics in culverts are poorly understood. Previous research indicated that bats with higher body mass at the onset of hibernation have a higher probability of surviving repeated arousal events from WNS. Therefore, we compared tricolored bat winter body mass between cave and culvert hibernacula and identified culvert characteristics influencing body mass during hibernation in Georgia, USA. From 2018 to 2022, we measured body mass of 754 individuals in early and late hibernation across 32 culverts (*n* = 497) and four caves (*n* = 257). Our study revealed a southward spread of the fungus over multiple years, with the first confirmed case of WNS in a Georgia culvert in 2022. Overall, tricolored bats in caves weighed more in early hibernation than those in culverts, but bats in culverts weighed more in late hibernation. Across all sites, female tricolored bats entering and leaving hibernation had greater mass than males but lost more mass during hibernation, possibly due to differences in torpor‐arousal patterns and WNS infection rates. Additionally, all bats lost more mass in longer culverts. Understanding culvert characteristics affecting bat body mass will inform management strategies to mitigate WNS effects. Identifying risk factors for specific tricolored bat hibernacula can guide managers on where to focus winter WNS monitoring efforts and potential treatments.

## Graphical abstract

We compared tricolored bat winter body mass between cave and culvert hibernacula and examined culvert characteristics that influence body mass during hibernation. Tricolored bats in caves weighed more in early hibernation than those in culverts, but bats in culverts weighed more in late hibernation. Across all sites, female tricolored bats entering and leaving hibernation had greater mass than males but lost more mass during hibernation. Understanding culvert characteristics affecting bat body mass will inform management strategies to mitigate white‐nose syndrome impacts.

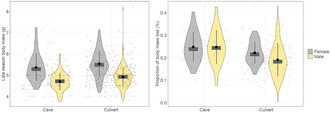

## 1 Introduction

White‐nose syndrome (WNS), a fungal disease caused by *Pseudogymnoascus destructans* (*Pd*), has resulted in significant mortality in multiple North American bat species (Lorch et al. 2011; Warnecke et al. 2012). The psychrophilic fungus presents as white fungal growth on the muzzle, ears, and/or wing membrane of infected bats and causes tissue erosion (Lorch et al. 2011; Blehert 2012). Mortality from WNS occurs as the fungal infection increases arousal frequency during hibernation, causing depletion of fat reserves and consequent death via starvation or dehydration (Blehert 2012; Reeder et al. 2012; Verant et al. 2014; Cheng et al. 2019). Hibernaculum mortality rates > 90% from WNS have been documented for multiple cave‐dwelling species in the northeastern and midwestern United States (Reeder et al. 2012; Langwig et al. 2015). Despite milder winters in the southeastern United States, significant disease‐related mortality, consistent with more northerly climates, has been observed in species such as tricolored bat ( *Perimyotis subflavus* ), little brown myotis ( *Myotis lucifugus* ), and northern long‐eared myotis ( *Myotis septentrionalis* ) (Jonasson and Willis 2011; Bernard et al. 2017; Czenze, Jonasson, and Willis 2017; Cheng et al. 2021; Perea et al. 2023).

Body condition is a key factor determining the ability of bats to survive WNS infection (Haase et al. 2021; Cheng et al. 2019). During hibernation, bats undergo torpor, reducing their metabolic rate and body temperature to conserve energy (Jackson, Willcox, and Bernard 2022). Torpor is periodically interrupted by arousal events, with a resultant increase in metabolic rate, temperature, and greater use of fat reserves (Ruf and Geiser 2015). The hibernation optimization hypothesis (Boyles et al. 2007) suggests bats balance the metabolic expense of hibernation through the advantageous selection of roosting microclimates (Willis 2017; Boyles et al. 2022). Bats with greater percent body fat at the beginning of hibernation experience increased survival rates (Brownlee‐Bouboulis and Reeder 2013; Cheng et al. 2019; Haase et al. 2021; Frick et al. 2022). The thrifty female hypothesis contends that although fat reserves are critical for bat survival during WNS infections, optimizing energy expenditure during winter is more important for female bats to successfully reproduce after hibernation (Jonasson and Willis 2011; Czenze, Jonasson, and Willis 2017). While females may survive WNS infection and its resultant effects of accelerated fat depletion during winter, they may experience sub‐lethal effects from disease‐influenced reductions to body condition, potentially impacting their ability to subsequently reproduce after spring emergence (Jonasson and Willis 2011; Meierhofer et al. 2018; Bernard et al. 2020).

The tricolored bat was formerly common in eastern North America, but populations have declined substantially due to WNS‐related mortality (Turner, Reeder, and Coleman 2011; Ingersoll, Sewall, and Amelon 2013; Frick et al. 2022). Cheng et al. (2021) reported that 59% of the tricolored bat's range has been impacted by WNS with a mortality rate of 93%, based upon reductions in winter colony abundance, prompting the U.S. Fish and Wildlife Service in 2022 to propose the tricolored bat as Endangered under provisions of the Endangered Species Act (U.S. Fish and Wildlife Service 2022). Most mortality has occurred in the core of the range, with observed population declines of up to 73% in the midwestern United States (Langwig et al. 2015). Declines in tricolored bat populations have also been observed in parts of the southeastern United States, which represents the eastern periphery of the species global range. Tricolored bat declines in the southeastern United States have been documented in surveys of overwintering populations (Loeb and Winters 2022; Perea et al. 2024) and in reduced summer activity determined via acoustic monitoring (Perea et al. 2022; Udell et al. 2022).

Although typically hibernating in caves or mines, when these features are absent, tricolored bats may use roadway culverts as winter hibernation sites (Keeley and Tuttle 1999; Lutsch 2019; Meierhofer, Johnson, et al. 2019; Meierhofer, Leivers, et al. 2019). Tricolored bats have been documented using culverts as hibernacula in many southeastern states, frequently in large numbers (Sandel et al. 2001; Katzenmeyer 2016; Stevens et al. 2017; Meierhofer, Johnson, et al. 2019; Meierhofer, Leivers, et al. 2019). Generally, bats select long, box‐shaped culverts with multiple boxes (i.e., individual culverts positioned immediately adjacent to one another) (Keeley and Tuttle 1999; Katzenmeyer 2016; Meierhofer, Leivers, et al. 2019), but culvert use also depends on landscape context and available microclimates within the structure (Meierhofer, Leivers, et al. 2019). Ambient roosting conditions experienced by bats within culvert hibernacula are influenced by external weather conditions and differ based on factors such as culvert dimensions, air flow, complexity, and distance of the roost from the inlet/outlet (Leivers et al. 2019).

In winter 2020, biologists with the Georgia Department of Natural Resources (GADNR) detected *Pd* on tricolored bats roosting in roadway culverts (Georgia Department of Natural Resources 2020). Given that body condition influences bats' ability to survive WNS, relating variables of hibernation bioenergetics and late hibernation body condition to winter roost choice is critical for understanding tricolored bat responses to *Pd* and implications for WNS pathophysiology. Therefore, our primary objectives were to compare tricolored bat winter body mass between caves and culverts and identify culvert attributes that influence body mass across Georgia, USA. A secondary objective was to document the spatiotemporal progression of *Pd* and WNS across the study area.

## 2 Methods

### 2.1 Study Area

**FIGURE 1:**
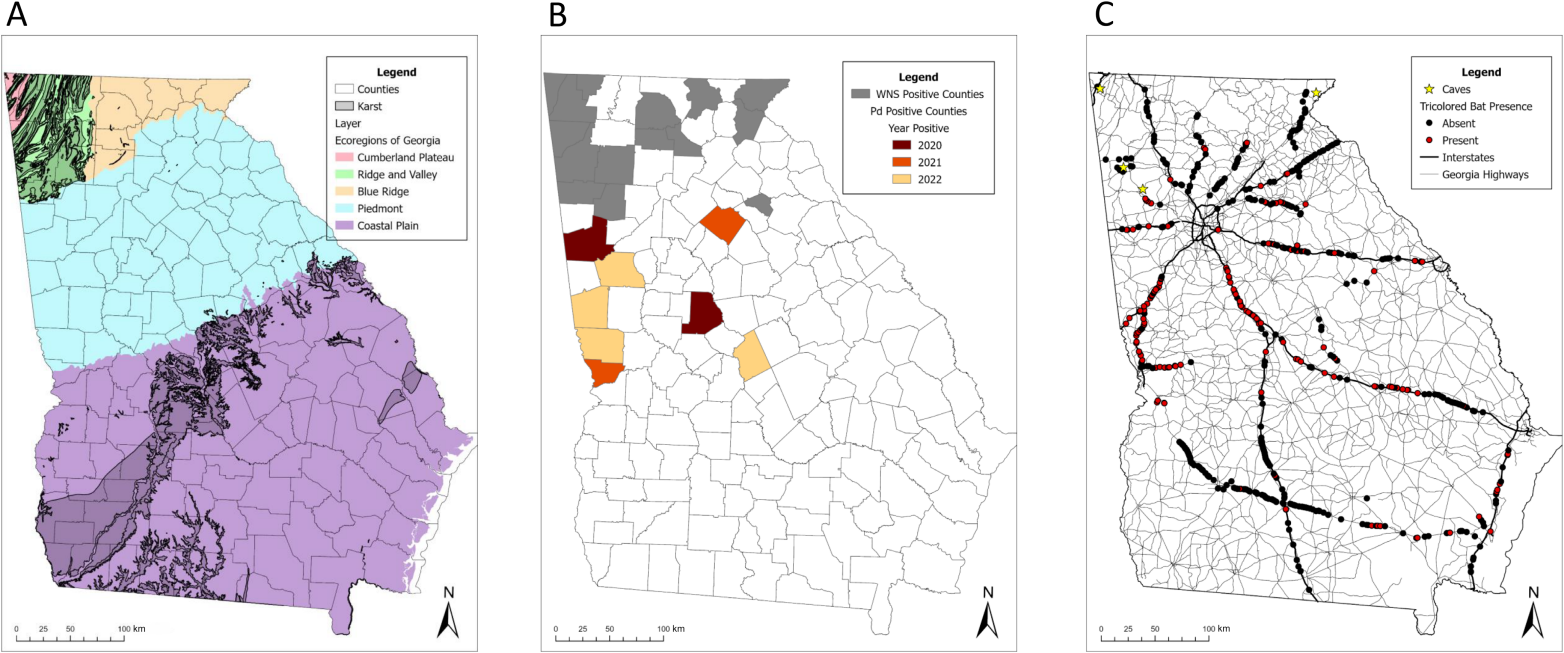
Level III ecoregions (Omernik and Griffith 2014) showing exposed karst areas (A; Weary and Doctor 2014), distribution of white‐nose syndrome (WNS) and *Pseudogymnoascus destructans* (*Pd*)‐positive counties by year of detection (B), and distribution of road culverts (*n* = 560) and caves (*n* = 4) surveyed for tricolored bats (C; *Perimyotis subflavus* ) in Georgia, USA, November–March, 2018–2022.

We conducted hibernaculum surveys in all ecoregions of Georgia, USA, across sites that included WNS‐positive areas and areas putatively naïve to *Pd* (Figure 1A,B; Edwards, Ambrose, and Kirkman 2013; Georgia Department of Natural Resources 2020). The Cumberland Plateau and Ridge and Valley ecoregions are typified by karst topography (Figure 1A), a high density of caves, and are dominated by mixed hardwood forests. The Blue Ridge consists of mountainous topography, is dominated by hardwood forests, and contains few caves. WNS has been detected in all three ecoregions for more than a decade (Figure 1B). The Piedmont ecoregion is composed of primarily oak (*Quercus* spp.), hickory (*Carya* spp.), and oak‐pine (*Pinus* spp.) forests, which have been heavily fragmented due to urban development. This region has few caves and was confirmed *Pd*‐positive in 2020 and WNS‐positive in 2022. The Coastal Plain has areas of exposed karst but is WNS‐negative, although a county along the northern edge of the region tested *Pd‐*positive in 2022. Land use in the Coastal Plain is dominated by agriculture, and the remaining upland forests are dominated by pines. All caves were in the Cumberland Plateau, Blue Ridge, and Ridge and Valley, and culverts were surveyed in all ecoregions.

### 2.2 Data Collection

We conducted hibernaculum surveys from November 2018 to March 2022 to quantify bat abundance and body mass. Caves were selected based on site accessibility and likelihood of bats being present based on previous surveys conducted by GADNR. We identified culverts along interstate and state highways using data from the Georgia Department of Transportation (Figure 1C), prioritizing long, box‐shaped culverts with multiple boxes, which have been shown to have greater tricolored bat abundance in previous research (Meierhofer, Leivers, et al. 2019). We selected culverts for study based on safe accessibility (i.e., large roadways with shoulders for parking) and for travel efficiency (i.e., multiple culverts along single stretches of road). Where known culvert locality was limited, we used Google Earth to identify potential survey areas. We located additional culvert sites opportunistically during fieldwork. For access and safety, we only surveyed culverts ≥ 0.9 m tall × 0.9 m wide that were safely wadable. We measured (m) width, height, and length during initial culvert visits using a laser distance finder (Bosch BLAZE 65‐ft Outdoor Laster Distance). A subset of 100 culverts received multiple visits (2–4 times/winter) to monitor temporal variation in bat colony abundance, body biometrics, and disease surveillance. Additionally, from 2019 to 2022, we visited caves twice/winter, once in early hibernation (November–December) and once in late hibernation (February–March) to collect bat biometric data.

At all culverts and caves surveyed, we located tricolored bats by searching all safely accessible areas. When tricolored bats were identified, we recorded body mass, age, sex, forearm length, visible fungal growth indicative of WNS, and wing damage score (Reichard and Kunz 2009) for ≤ 20 bats/visit/site. Males were categorized as scrotal or non‐scrotal (Racey 2009). We chose a maximum of 20 bats/site due to time constraints and to limit colony disturbance. Bats were banded (2.4 mm Porzana metal lipped band) using banding pliers prior to release. At sites with standing or flowing water where there was a risk of bats falling or flying into the water after release, a circulated air incubator (Genesis Hova‐Bator, GQF Manufacturing, Savannah, Georgia, USA) was used to assist bats in reaching euthermy. Upon site revisits, we counted the number of banded bats and prioritized recapturing those bats for biometric measures to quantify mass loss. While we were aware that our presence could potentially disturb bats not included in the captured sample, arousal of uncaptured tricolored bats was rare.

To determine *Pd* presence, we swabbed a maximum of 5 bats/culvert in late winter (February–March). The maximum number of swabs was an effort to sample as many culverts as possible across the study area given budgetary constraints. We limited sampling to one representative culvert/county, and only known or suspected *Pd‐*negative counties were included. Bat swabs were taken by rolling a cotton‐tipped swab five times along the length of the forearm and five times on the muzzle. When possible, up to five environmental surface swabs/site were taken within 3 m of known or suspected bat roosts by rolling the swab 10 times on the wall/ceiling surface. All swabs were collected, maintained, and transported according to the most current guidelines from the National WNS Disease Surveillance Working Group (https://www.whitenosesyndrome.org/working‐group/surveillance‐and‐diagnostics). Analysis of swab samples for determination of *Pd* presence was conducted at the BioInnovation Laboratory at Kennesaw State University according to current methods approved by the Disease Surveillance Working Group using the Muller et al. (2013) quantitative polymerase chain reaction (qPCR) technique.

Additional culverts (two in winter 2019, six in winter 2020, seven in winter 2021, and two in winter 2022) were sampled by GADNR as a part of annual WNS monitoring. Those sampling events adhered to protocols established by the National Wildlife Health Center (NWHC), and *Pd* swabbing methodology for these samples was the same as previously described. Bats swabbed (≤ 25/site) were selected based on accessibility and to ensure an even distribution throughout the roost. No body mass data were collected. When 25 bats could not be sampled, two environmental samples were taken for every bat sample not collected to ensure adequate *Pd* sampling across the site to monitor for possible fungal presence.

We conducted decontamination procedures for all cave surveys, and between culverts > 32 km apart, following the National White‐nose Syndrome Decontamination Protocol (White‐nose Syndrome Disease Management Working Group 2018). For surveys that occurred during the SARS‐CoV‐2 global pandemic, we adhered to mitigation measures for bat safety as recommended by the International Union for Conservation of Nature (IUCN) Species Survival Commission Bat Specialist Group (Kingston et al. 2021). All survey, capture, and handling methods were conducted under Animal Use Protocol number A2020 03‐021‐Y1‐A0 approved by the University of Georgia's Institutional Animal Care and Use Committee. Current Georgia Department of Natural Resource staff, including co‐author Emily Ferrall, are covered under a state scientific collection permit as employees of the Wildlife Resources Division, Wildlife Conservation Section of GADNR. GADNR employees are permitted to work with listed federal species under a Cooperative Agreement under Section 6 of the Endangered Species Act between the U.S. Fish and Wildlife Service and GADNR.

### 2.3 Statistical Analysis

All analyses were performed in R version 4.1.1 (R Development Core Team 2020). We used total body mass as a measure of body condition because it is as effective as other common body condition indices for estimating fat reserves and does not require specialized equipment to obtain (McGuire et al. 2018). We examined the influence of sex and hibernaculum type as drivers of mass loss by evaluating the proportion of mass lost from banded individuals (i.e., early hibernation mass minus late hibernation mass, divided by early‐season mass). We pooled mass lost across individual bats within caves and culverts for comparison. Because of unequal population variances, we used Welch's *t*‐tests to compare late hibernation (February–March) body mass and proportion of body mass lost for each sex by hibernacula type. We evaluated relationships between late hibernation body mass and proportion of body mass lost in culverts against a suite of predictor variables (Table 1) using generalized linear mixed‐effects models (GLMM) via package glmmTMB (Brooks et al. 2017). We developed candidate model sets containing individual variables, combinations, and interactions of variables, a global and a null model (intercept‐only) with site as a random effect in all models. For late hibernation body mass as the response variable, we built nine candidate models that included sex, survey year, and latitude. We used Gaussian regression because the response variable was a continuous numerical value. For proportion of body mass lost over winter as the response variable, we built 23 models that included sex, survey year, latitude, culvert length, width, and height. We examined proportion of body mass loss as a function of covariates using beta regression due to its ability to handle bounded data (e.g., proportions and percentages that are constrained between 0 and 1). For both analyses, we specified females as the reference group. We tested for correlation among continuous predictor variables using Pearson's correlation coefficient to ensure that highly correlated (*r* ≥ |0.7|) variables were not included in the same model. We used Akaike's Information Criterion corrected for small sample size (AICc) to calculate Akaike's model weights (*w*_*i*_) and determine the most parsimonious model(s) (Burnham and Anderson 2002). We considered models < 2 ΔAICc units from the top model to be equally informative. We evaluated the best‐supported models for goodness‐of‐fit and over‐ and under‐dispersion using a QQ plot, residual plot, and one‐sample Kolmogorov–Smirnov test with the DHARMa package in R (Hartig 2020). While we focused on the top model of each set following Arnold (2010) to avoid potential problems with model averaging (Cade 2015), we considered variables not included in the top model but with a *p*‐value < 0.05 as influential.

**TABLE 1:** Variables potentially influencing late body mass and proportion of body mass lost, hypothesized direction of influence, and predicted effects for culvert hibernating tricolored bat ( *Perimyotis subflavus* ) in Georgia, USA, November–March, 2018–2022.

Covariate	Direction	Prediction
Late hibernation body mass (g)		
Sex	+	Female bats will weigh more in late hibernation than males
Survey year	±	Body mass will vary based on yearly environmental conditions
Latitude	+	Culvert hibernating bats at higher latitudes will have a greater body mass in later hibernation than those at lower latitudes
Body mass loss (g)		
Sex	+	Female bats will lose less body mass during hibernation than males
Survey year	±	Body mass loss will vary based on annual environmental conditions
Latitude	−	Culvert hibernating bats at higher latitudes will lose more body mass during hibernation than those at lower latitudes
Culvert length	−	Bats in longer culverts will lose less body mass than those in shorter culverts
Culvert width	+	Bats in narrower culverts will lose less body mass than those in wider culverts
Culvert height	+	Bats in shorter culverts will lose less body mass than those in taller culverts

## 3 Results

Tricolored bats were present at all four caves and 173/560 (30.9%) culverts surveyed (Figure 1C). Mean culvert length, width, and height were 117.5 m (43.0–312.1 m), 2.4 m (1.2–2.7 m), and 2.1 m (1.2–3.1 m), respectively. We collected biometric data and/or *Pd* swabs from 754 unique individuals (261 female, 493 male) from culvert and cave hibernacula. We recaptured 241 (32.0%) unique banded individuals (26.6% caves, 34.7% culverts) in subsequent years upon repeated site visits. We collected late hibernation body mass on 477 bats. Of the 198 bats sampled twice within a single winter season to obtain proportion of mass loss, 62 were from caves and 136 from culverts (61 female and 137 male).

All caves surveyed were *Pd‐* and WNS‐positive. We observed a spatiotemporal progression of *Pd* in culvert hibernacula, with three counties documented as *Pd‐*positive in winter 2020, two in 2021, and six in 2022, totaling 11 *Pd‐*positive counties during the study period (Figure 1B). In winter 2022, we observed a clinical field sign consistent with WNS on hibernating tricolored bats at a culvert that had been confirmed as *Pd‐*positive in winter 2020. Samples were taken and a WNS diagnosis was verified by the National Wildlife Health Center via histopathology.

**FIGURE 2:**
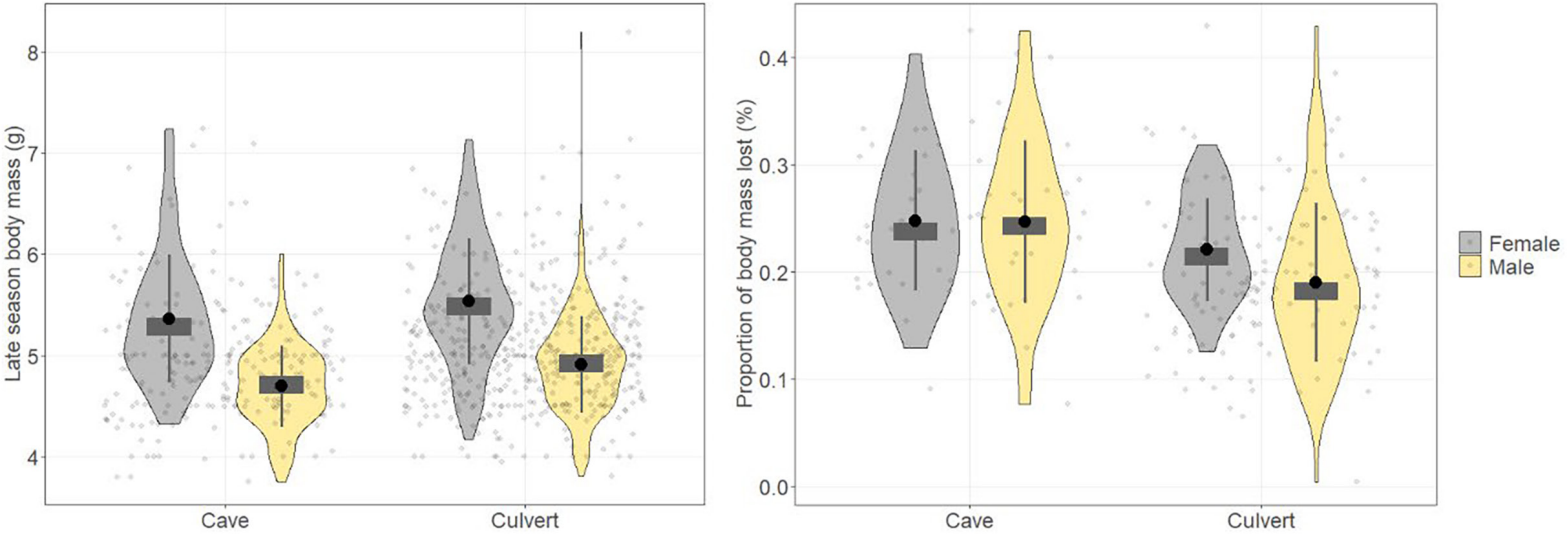
Late hibernation body mass (g) and proportion of body mass lost in male and female tricolored bats ( *Perimyotis subflavus* ) in caves and culverts in Georgia, USA, November–March, 2018–2022. Density plots show data distribution, dark gray vertical lines show interquartile ranges, dark gray rectangles indicate the median, and black circles indicate the mean. Width of plots adjusted for visualization purposes.

Female (*t*_167_ = 2.84, *p* = 0.005) and male (*t*_315_ = 4.62, *p* < 0.001) early hibernation mass was greater in caves than culverts (Table 2). Late hibernation body mass of females (*t*_225_ = −1.98, *p* = 0.049), males (*t*_489_ = −5.17, *p* < 0.001), and both sexes combined (*t*_714_ = −3.01, *p* = 0.003) was greater in culverts than in caves (Figure 2A). For both sexes combined, the proportion of body mass lost was greater (*t*_198_ = 4.60, *p* < 0.001) in caves than in culverts. Males in culverts lost less (*t*_137_ = 3.85, *p* < 0.001) mass than those in caves, whereas female mass loss was similar (*t*_61_ = 1.81, *p* = 0.077) between caves and culverts (Figure 2B).

**TABLE 2:** Mean (SD) early and late hibernation body mass and proportion mass loss for females, males, and bats of both sexes combined in cave and culvert hibernacula surveyed in Georgia, USA, November–March, 2018–2022.

Covariate	Cave			Culvert		
	Female	Male	Combined	Female	Male	Combined
Early hibernation mass	7.12 (0.71)	6.39 (0.68)	6.74 (0.78)	6.82 (0.65)	6.01 (0.56)	6.24 (0.69)
Late hibernation mass	5.36 (0.63)	4.69 (0.40)	4.95 (0.60)	5.53 (0.62)	4.91 (0.47)	5.09 (0.59)
Proportion mass loss	0.25 (0.07)	0.19 (0.07)	0.25 (0.07)	0.22 (0.05)	0.19 (0.17)	0.20 (0.07)

The top model explaining late hibernation body mass in culverts included only sex as a covariate (Table 3), with females weighing more than males (Figure 2, Table 4). There were two additional models with ΔAICc < 2, containing the variables sex, year, and latitude (Table 3); however, sex was the only covariate significant in all three models (Table A1 in Appendix 1). For proportion of mass lost, the top model contained the covariates sex and culvert length (Table 3), indicating that females lost more mass than males and that bats lost more mass as culvert length increased (Table 4). There were four additional models with ΔAICc < 2 that included the covariates sex, year, sex by year interaction, latitude, culvert length, and culvert width. Sex was present and an influential predictor in all models (*p* < 0.05, Table 4). Culvert length was included and an influential predictor (*p* < 0.05, Table 4) in three models. No other variables were found to be statistically significant (Table A1 in Appendix 1).

**TABLE 3:** Top models (≤ 2 ΔAICc) for predicting late‐season (February–March) tricolored bat ( *Perimyotis subflavus* ) body mass (g) and proportion of body mass loss in culverts located in Georgia, USA, November–March, 2018–2022.

Model	*K*	AICc	ΔAICc	*ω*_*i*_
Late hibernation body mass (g)				
~sex + (1|site)	4	731.01	0	0.37
~sex + year + latitude + (1|site)	6	731.27	0.26	0.33
~sex + latitude + (1|site)	5	731.47	0.46	0.30
Body mass loss				
~sex + length + (1|site)	5	−345.74	0	0.21
~sex + (1|site)	4	−344.96	0.78	0.14
~sex*year + length + (1|site)	7	−344.79	0.95	0.13
~sex + length + width + (1|site)	6	−343.87	1.87	0.08
~sex + latitude + (1|site)	5	−343.79	1.95	0.08

*Note:* Table includes number of parameters (*K*), Akaike's Information Criterion corrected for small sample size (AICc), difference between a model and the model with the lowest AICc value (ΔAICc), and model weight (*ω*_*i*_). Site was included as a random effect in all models, and female was designated as the reference group for the sex covariate.

**TABLE 4:** Top model estimated coefficients for predicting tricolored bat ( *Perimyotis subflavus* ) late hibernation body mass and proportion of body mass loss in Georgia culverts, USA, November–March, 2018–2022.

Model	Covariate	Estimate	SE	*Z*	*p*
Late hibernation body mass (g)					
~sex + (1|site)	Intercept	5.54	0.05	107.38	< 0.001
	Sex (M)	−0.61	0.05	−11.44	< 0.001
Body mass loss					
~sex + length + (1|site)	Intercept	−1.25	0.08	−15.00	< 0.001
	Sex (M)	−0.22	0.08	−2.66	0.008
	Length	0.13	0.06	2.10	0.035

*Note:* Female was designated as the reference group for the sex covariate.

## 4 Discussion

Although *Pd* had been previously documented in culvert hibernacula (Bernard et al. 2019; Meierhofer, Leivers, et al. 2019), uncertainty persisted if environmental conditions would provide conditions for *Pd* to present clinically as WNS (Leivers et al. 2019; Meierhofer, Johnson, et al. 2019). With our confirmed WNS case in 2022, in concert with a documented case in a culvert‐roosting tricolored bat in Mississippi in the same year (White‐nose Syndrome Response Team 2022), development of WNS in culverts is a noteworthy discovery. Our observation of disease presentation in a culvert where *Pd* detection occurred 2 years prior is consistent with observations in cave hibernacula where WNS pathophysiology is typically delayed 1–5 years after *Pd* arrival (Bernard and McCracken 2019; Frick et al. 2017).

Cave hibernating bats weighed less during late hibernation and lost proportionally more mass, likely due to confounding effects of cave location, WNS status, and hibernaculum structure that influence external weather and within‐roost microclimate conditions. Because caves in our study were in the northern portion of the state, the hibernation period is likely prolonged compared to culvert hibernacula, most of which occurred south of the mountainous ecoregions. Therefore, cave hibernating bats in our study area may enter hibernation earlier and use more fat stores compared to those in culverts. Bats inhabiting southern latitudes have opportunities to feed later into the fall, or to replenish fat stores lost mid‐winter via nighttime foraging (Bernard et al. 2021; Frick et al. 2022; Perea et al. 2024). Additionally, bats hibernating in warmer conditions use ambient conditions to arouse from torpor (Boyles et al. 2007), which may lower energy and body mass expenditure for culvert hibernating tricolored bats. WNS clinical field signs were observed in all cave hibernacula (Perea et al. 2024); therefore, cave bats were more likely to incur repeated arousals that caused increased mass loss (Storm and Boyles 2011; Reeder et al. 2012). The differences in late hibernation mass and proportion of mass loss also could be related to differences in microsite conditions between caves and culverts. Boyles et al. (2022) found that tricolored bats selected warm, humid microclimates, indicating that roost selection is a critical behavioral tactic for energy conservation. Although we did not measure humidity, based on Leivers et al. (2019), culverts may have lower humidity which potentially impacts torpor‐arousal patterns and consequently body condition. Conversely, Loeb and Winters (2022) found that tricolored bats in a WNS‐positive tunnel selected colder microclimates, suggesting that slower *Pd* growth rates or lower energetic costs associated with colder temperatures may be advantageous.

We observed that female tricolored bats had greater late hibernation body mass regardless of hibernaculum type. Bats with greater body mass have more energy to use during periods of arousal to overcome costs of hibernation and potential negative effects on reproductive success upon spring emergence (Meierhofer et al. 2018; Boyles et al. 2020). Although greater mass in females seems to support the thrifty female hypothesis (Jonasson and Willis 2011), our observation of females in culverts losing a greater proportion of body mass compared to males is counter to the hypothesis. Similarly, in a controlled laboratory study, McGuire, Johnson, et al. (2021) observed that female tricolored bats lost more mass than males during hibernation and theorized that lack of conformation to the hypothesis was due to geographic location of the studies. The research that led to the thrifty female hypothesis occurred in Canada where winters are harsher and hibernation may be extend over longer periods. Similar to McGuire, Johnson, et al. (2021), our study occurred at the southern extent of the tricolored bat range where milder winter conditions likely allow for a different life history strategy. Bats at southern latitudes experience a shorter hibernation period given differences in winter climates compared to northern latitudes and are often provided opportunities to forage mid‐winter, resulting in them being less constrained by initial fat reserves to survive the winter (Bernard and McCracken 2019; Bernard et al. 2021; Perea et al. 2024).

The greater mass loss we observed in culvert‐roosting female tricolored bats compared to males is likely due to behavioral and physiological differences between sexes (Kailing et al. 2023). Bats are often active during winter in the southeastern United States (Perea et al. 2023). Males may be more likely to exhibit foraging activity in winter (Avery 1985), allowing them to replenish fat reserves. Alternatively, females may use microclimates within culverts that are less thermally constant or humid than males. Roosting in less stable thermal conditions and in areas of low moisture has been shown to increase body mass loss of female tricolored bats (McGuire, Johnson, et al. 2021). Leivers et al. (2019) found that culvert microclimates are influenced more by external weather conditions than caves and these factors determine bat site use (Meierhofer, Leivers, et al. 2019). Given that microclimate conditions in hibernacula play a major role in disease severity of WNS‐infected individuals, with warm and humid conditions increasing fungal development (Langwig et al. 2012, 2021; Haase et al. 2021; Hoyt, Kilpatrick, and Langwig 2021; Frick et al. 2022), understanding how these roost site variations impact body mass is imperative to species conservation.

The positive relationship observed between mass loss and culvert length is counter to our original hypothesis. We expected that longer culverts would be more thermally stable, as suggested by Meierhofer, Leivers, et al. (2019), leading to fewer arousals, ultimately resulting in less mass loss. However, mean length of all culverts in our study was 2.5 times greater than mean culvert length in Meierhofer, Leivers, et al. (2019). In caves, variation in interior and external air temperatures can result in thermal convection, which often occurs as a “chimney effect” in which air flows in or out to reach a state of equilibrium, potentially resulting in near constant airflow in sites with greater temperature dissimilarity (Perry 2013). Longer culverts like those in our study may experience a “chimney effect” similar to caves that can cause increased airflow. Greater airflow increases evaporation rates (Perry 2013). Evaporative water loss during hibernation is considered a primary cause of WNS bat mortality as bats suffer from dehydration, or secondary effects of increased arousal frequencies required to replenish water loss that can negatively impact body mass conservation (Cryan et al. 2010; Perry 2013). Tricolored bats are highly susceptible to evaporative water loss during hibernation (McGuire, Fuller, et al. 2021), resulting in selection of humid microclimates (Frick et al. 2022). Greater evaporative water loss in longer culverts due to the increased airflow may impact torpor‐arousal patterns that alter winter bat mass loss, which would coincide with our observation of tricolored bats losing more mass with increasing culvert length.

Despite our study occurring across a latitudinal gradient, latitude was not an important predictor of late hibernation body mass in culvert hibernacula. The greatest proportion of used culvert hibernacula occurred in the Piedmont and upper portion of the Coastal Plain, both of which experience relatively mild winter temperatures. Thus, we suggest that there may not have been sufficient temperature variation to influence body mass in culverts. Mild winter temperatures permit bat activity on warm nights, allowing bats to replenish energy lost from arousals (Grider et al. 2016; Parker et al. 2020; Bernard et al. 2021; Andersen et al. 2022; Perea et al. 2023). Additionally, tricolored bats in southern latitudes may maintain lower fat reserves due to shorter hibernation periods and compensate for mass loss behaviorally by opportunistically foraging during winter (Lacki et al. 2015). Frick et al. (2022) suggested that there may be a regional refugia effect that reduces WNS impacts on southern tricolored bat populations by their intermittently feeding to replenish fat stores. Bernard and McCracken (2019) found that bats in the southeastern United States use periods of warm weather to feed despite *Pd* impacts. In concurrence with these studies, our results support the theory that tricolored bats at lower latitudes can afford to weigh less because they experience more frequent warm periods during which they can replenish fat stores, in contrast to bats further north that must carry increased mass to survive longer periods of inactivity.

In summary, for both sexes, we found late hibernation body mass was greater in culverts than caves and that bats in caves lost a greater proportion of body mass during hibernation. Given that all cave hibernacula were WNS‐positive (Perea et al. 2024), whereas WNS was only observed in one culvert, these differences are likely due to repeated arousals of bats in caves caused by WNS. Greater loss of body mass in caves also may be confounded by the location of caves at more northern latitudes and the concomitant climatic differences. For culvert hibernating bats, sex had a significant influence on late hibernation body mass with females weighing more than males upon emergence. Sex also appears to be an important factor influencing proportion of mass loss overwinter, with females losing more mass, possibly due to sex‐specific behavioral and microclimate roost site selection differences. Although the mechanism driving greater mass loss in longer culverts is not fully understood, evaporative water loss is suspected to play a role.

Importantly, we documented that tricolored bats hibernating in culverts can develop WNS. Thus, our research contributes to a greater understanding of culvert characteristics affecting the bat body condition that will inform management strategies to mitigate WNS impacts. Our results provide baseline information for identifying risk factors in culvert hibernacula that can guide managers on where to focus WNS monitoring efforts. Furthermore, if effective treatment strategies are developed, our results can be used to target culverts where treatment will provide the most benefit and most efficient use of resources (Bernard et al. 2019). For example, longer culverts, which are associated with greater mass loss and potentially higher mortality in WNS‐infected bats, could be prioritized for treatment. Future research that examines a greater range of culvert types and dimensions could clarify the mechanism(s) driving greater mass loss in longer culverts.

## Author Contributions

**Emily A. Ferrall:** conceptualization (equal), data curation (lead), formal analysis (lead), investigation (lead), methodology (equal), writing – original draft (lead), writing – review and editing (equal). **Santiago Perea:** formal analysis (equal), software (equal), visualization (equal), writing – review and editing (equal). **Katrina M. Morris:** conceptualization (equal), funding acquisition (supporting), investigation (supporting), methodology (equal), project administration (supporting), resources (supporting), supervision (equal), writing – review and editing (equal). **Pete E. Pattavina:** conceptualization (equal), funding acquisition (supporting), investigation (supporting), project administration (supporting), writing – review and editing (supporting). **Brian J. Irwin:** conceptualization (equal), formal analysis (equal), writing – review and editing (equal). **Jeff Hepinstall‐Cymerman:** conceptualization (equal), formal analysis (supporting), methodology (supporting), software (supporting), writing – review and editing (equal). **Steven B. Castleberry:** conceptualization (equal), formal analysis (supporting), funding acquisition (lead), project administration (lead), resources (supporting), supervision (equal), writing – original draft (supporting), writing – review and editing (equal).

## Conflicts of Interest

The authors declare no conflicts of interest.

## Supporting information


Data S1.

## Data Availability

All of the data required for reproducing the results of the present study are uploaded as Data S1.

## Appendix 1

**TABLE A1:** Models within 2 ΔAIC_c_ and estimated coefficients for predicting tricolored bat ( *Perimyotis subflavus* ) late hibernation body mass and proportion of body mass lost in Georgia USA, culverts, November–March, 2018–2022.

Model	Covariate	Estimate	SE	*Z*	*p*
Late xhibernation body mass (g)					
~sex + (1|site)	Intercept	5.54	0.05	107.38	< 0.001
	Sex (M)	−0.61	0.05	−11.44	< 0.001
~sex + year + latitude + (1|site)	Intercept	5.54	0.05	107.57	0.157
	Year	0.04	0.03	1.50	0.134
	Sex (M)	−0.61	0.05	−11.42	< 0.001
	Latitude	−0.03	0.03	−0.97	0.332
~sex + latitude + (1|site)	Intercept	5.53	0.05	110.60	< 0.001
	Sex (M)	−0.61	0.05	−11.37	< 0.001
	Latitude	−0.04	0.03	−1.35	0.177
Body mass loss					
~sex + length + (1|site)	Intercept	−1.25	0.08	−15.00	< 0.001
	Sex (M)	−0.22	0.08	−2.66	0.008
	Length	0.13	0.06	2.10	0.034
~sex + (1|site)	Intercept	−1.28	0.10	−13.46	< 0.001
	Sex (M)	−0.22	0.09	−2.56	0.010
~sex*year + length + (1|site)	Intercept	−1.27	0.08	−15.62	< 0.001
	Sex (M)	−0.23	0.08	−2.68	0.007
	Year	0.03	0.07	0.35	0.726
	Length	0.12	0.06	2.12	0.034
	Sex*Year	−0.11	0.08	−1.33	0.185
~sex + length + width + (1|site)	Intercept	−1.24	0.08	−15.13	< 0.001
	Sex (M)	−0.23	0.08	−2.67	0.008
	Length	0.14	0.06	2.24	0.030
	Width	0.03	0.05	0.57	0.566
~sex + latitude + (1|site)	Intercept	−1.26	0.09	−13.62	< 0.001
	Sex (M)	−0.22	0.09	−2.57	0.010
	Latitude	−0.07	0.07	1.04	0.299

*Note:* Culvert was included as a random effect in all models. Female was designated as the reference group for the sex covariate.

Abbreviation: AICc, Akaike's Information Criterion corrected for small sample size.
